# Partial weight support of the arm affects corticomotor selectivity of biceps brachii

**DOI:** 10.1186/s12984-015-0085-6

**Published:** 2015-10-26

**Authors:** Keith D. Runnalls, Greg Anson, Winston D. Byblow

**Affiliations:** Movement Neuroscience Laboratory, University of Auckland, Auckland, New Zealand; Centre for Brain Research, University of Auckland, Auckland, New Zealand

**Keywords:** Gravity compensation, Arm weight support, Motor cortex, Transcranial magnetic stimulation, Integrated control, Selective activation

## Abstract

**Background:**

Weight support of the arm (WS) can be used in stroke rehabilitation to facilitate upper limb therapy, but the neurophysiological effects of this technique are not well understood. While an overall reduction in muscle activity is expected, the mechanism by which WS may alter the expression of muscle synergies has not been examined until now. We explored the neurophysiological effect of WS on the selectivity of *biceps brachii* (BB) activation in healthy adults.

**Methods:**

Thirteen participants completed counterbalanced movement tasks in a repeated measures design. Three levels of WS (0, 45, and 90 % of full support) were provided to the arm using a commercial device (Saebo Mobile Arm Support). At each level of WS, participants maintained a flexed shoulder posture while performing rhythmic isometric elbow flexion (BB agonist) or forearm pronation (BB antagonist). Single-pulse transcranial magnetic stimulation of primary motor cortex was used to elicit motor-evoked potentials (MEPs) in BB 100–300 ms before muscle contraction. Baseline muscle activity and MEP amplitude were the primary dependent measures. Effects of movement task and support level were statistically analyzed using linear mixed effects models.

**Results:**

As expected, with increased support tonic activity was reduced across all muscles. This effect was greatest in the anti-gravity muscle *anterior deltoid*, and evident in *biceps brachii* and *pronator teres* as well. For BB MEP amplitude, task and support level, interacted such that for elbow flexion, MEP amplitudes were smaller with incrementally greater WS whereas, for forearm pronation MEP amplitudes were smaller only at high WS.

**Conclusions:**

Weight support of the arm influences corticomotor selectivity of *biceps brachii*. WS may impact coordination independently of a global reduction in muscle activity. The amount of supportive force applied to the arm influences the neuromechanical control profile for the limb. These findings may inform the application of WS in upper limb stroke rehabilitation.

## Background

Weight support of the arm (WS) can be used during stroke rehabilitation therapy to reduce difficulty, and increase the quality and quantity of movements made by patients with upper limb impairment [1–3]. A variety of devices, ranging from spring-based supports to robotic systems, have been employed to fully or partially compensate for the weight of the arm [4]. By lessening the magnitude of antigravity torques required for the performance of gross functional movements, WS improves execution of individual movements and may facilitate movement repetition. For example, WS mitigates the reductions in reaching work area observed in stroke patients with upper limb impairment [5]. Generally, WS is thought to benefit upper limb rehabilitation primarily by increasing capacity in terms of intensity or volume of therapeutic exercises [6].

While the dosage of training is a critical factor driving use-dependent plasticity and adaptive cortical reorganization [7–9], little is known about what patterns of neuromuscular activity are being expressed and learned under gravity compensated conditions. The application of WS can immediately reduce abnormal joint torque coupling between the shoulder and elbow, permitting hemiparetic individuals a greater range of elbow extension during forward reaching tasks [10, 11]. The effects of WS on movement kinematics are related to an overall reduction in the muscle activity needed to perform reaching tasks, which is evident in both healthy older adults and chronic stroke patients [12, 13]. Although it is apparent that WS can influence motor behavior of the upper limb, the mechanisms by which WS influences intra-limb coordination at the neural level remain unclear.

We previously examined the effects of WS on muscle activation and corticomotor excitability to proximal and distal upper limb muscles using motor evoked potentials from transcranial magnetic stimulation (TMS) in healthy adults [14]. As expected, tonic activity in the *anterior deltoid* (AD) responded linearly to WS. However, a modulation of tonic activity in more distal muscles indicated that WS also interacted with proximal-distal neural linkages. Additionally, corticomotor excitability (CME) to distal muscles was modulated by WS. In the forearm muscle *extensor carpi radialis*, CME decreased with the application of any WS. A different pattern of modulation was observed in the *first dorsal interosseous* of the hand, where CME increased, but only at a high level of WS. Nonlinear muscle-dependent CME responses suggest that under static conditions, the neural linkages with which WS interacts are not generalized across the limb and involve both excitatory and inhibitory mechanisms. Here we examine how WS impacts coordination *via* CME modulation in the context of movement.

The neurophysiological mechanisms of selective muscle recruitment can be examined using TMS [15–18]. In healthy adults, motor evoked potentials (MEPs) elicited in the antagonist *biceps brachii* (BB) are suppressed at a cortical level prior to forearm pronation. In contrast, suppression of BB MEPs is drastically reduced or absent in stroke patients with more severe upper limb impairment [16, 19]. The ratio of MEP amplitude preceding forearm pronation relative to the amplitude preceding elbow flexion has been shown to be correlated with upper limb impairment [16, 18]. This selectivity ratio can be interpreted as a neurophysiological measure of upper limb coordination that is sensitive to the coupling of elbow flexion and shoulder abduction that typifies the abnormal flexor synergy [10, 20]. The effects of WS on selective muscle recruitment and suppression of antagonist muscles may provide insights into the underlying pathophysiology of dysfunctional synergies and inform the clinical use of WS.

In the present study, we sought to examine the neurophysiological effect of WS on the selectivity of BB activation in healthy adults using TMS. We expected that increased WS would modulate isometric activity of AD and improve the selectivity of BB by reducing CME of BB preceding an antagonist contraction. We investigated parametric WS using a commercially available rehabilitation device that provided gravity compensation through a forearm brace. As a reference, tonic background activity was analyzed from the AD and BB muscles. We then examined CME of BB preceding phasic agonist (elbow flexion) and antagonist (forearm pronation) contractions by analyzing MEP amplitude. We hypothesized that an increase in WS would lead to a decrease in CME of BB preceding forearm pronation.

## Methods

### Participants

Fifteen right handed healthy adults (mean age: 23.8 years, range: 19.9–30.3 years, 9 female) without history of upper limb injury or neurological illness participated in this study. All procedures were approved by the University of Auckland Human Participants Research Ethics Committee in accordance with the Declaration of Helsinki. Participants provided written informed consent and were screened for contraindications to TMS by a neurologist.

### Design

Participants completed all experimental conditions in a single-session repeated measures design. Single-pulse TMS was used to elicit MEPs in BB during 2 rhythmic motor tasks (elbow flexion or forearm pronation) at 3 levels of WS (low, medium, high). The order of WS was counterbalanced across participants. Within each WS level, the elbow flexion task was always completed before the pronation task. The session lasted approximately 2.5 hours.

### Posture and arm support

**Fig. 1:**
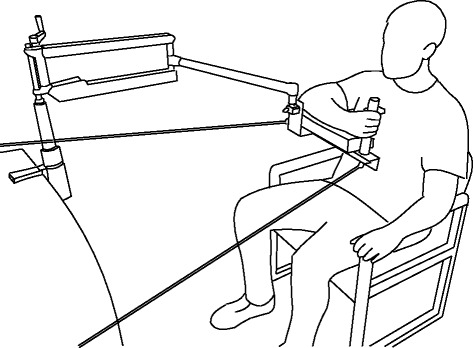
A schematic illustration of the experimental setup. The EMG electrodes and the elastic wrap used to secure the forearm to the brace have been omitted for clarity. A laser pointer attached to the brace provided visual feedback of the arm position

Figure 1 schematically illustrates the experimental setup. Participants were seated with their left arm resting on their lap. The right arm was supported by a mobile arm support system (SaeboMAS, Saebo Inc., Charlotte, NC). The SaeboMAS attaches to the forearm *via* a custom brace through which WS is provided and adjusted *via* spring tension. In the task, the brace was modified to also include a vertical cylindrical handle for participants to grasp, and a cushioned support surface for the elbow and forearm. The forearm was firmly secured to the brace using elasticized fabric wrap. Motor tasks were performed in a standardized arm posture with the shoulder flexed approximately 80° in the sagittal plane and internally rotated 90°. The elbow was flexed at 90° and the forearm supinated. The handle was grasped with the palm facing the torso using a neutral wrist position. Joint angles were set using a goniometer. The SaeboMAS prevented rotation of the brace in the vertical plane thus ensuring the forearm was always parallel to the floor. The brace was tethered to wall-mounted anchors using two nylon cords. This provided static resistance for the elbow flexion task and maintained a constant distance between the forearm and torso. The overall effect of the bracing and tethering was to enable isometric elbow flexion and forearm pronation tasks without restricting shoulder circumduction. Participants received visual feedback about their arm posture by centering a laser pointer on a circular target.

We defined three discrete levels of WS. At low support (0 %), the device compensated for its own weight and provided no further support. At medium and high support levels, the device provided 45 and 90 % of the force required to fully compensate for the weight of the arm. These values were determined from a force-titration procedure in which we monitored activity in the AD muscle and incrementally decreased the supportive force from a high setting until root mean squared EMG (rmsEMG) was observed to deflect away from baseline, and then scaled accordingly [14].

### Motor tasks

While maintaining the standardized posture using voluntary AD activity, participants performed either repetitive elbow flexion or forearm pronation tasks paced at 0.8 Hz by an auditory metronome. Participants were instructed to completely relax the task agonist between each contraction. Visual feedback of rectified EMG from the agonist was presented to participants to assist timing and relaxation. Three familiarization sets of each task were completed before collection. Data from the last familiarization set were averaged to obtain a time value for typical EMG burst onset relative to the metronome. Following familiarization and adjustments, 8 sets of contractions were collected for each condition. Each set consisted of 39 repeated contractions and was 49 s in duration. Adequate rest was provided between sets.

### Electromyography and transcranial magnetic stimulation

Surface electromyography was used to record activity from the right *anterior deltoid* (AD), *biceps brachii* (BB), and *pronator teres* (PT) muscles. Following skin preparation, self-adhesive 10 mm diameter Ag-AgCl electrodes (BlueSensor N; Ambu, Denmark) were arranged in a bipolar montage approximately 2 cm apart over the belly of each muscle. A common ground electrode was placed over the acromion process (Red Dot: 3 M Health Care, Canada). Signals were amplified (CED 1902; Cambridge Electronic Design, Cambridge, UK) with 1000× gain, band-pass filtered (5–1000 Hz), sampled at 2 kHz (CED 1401), and saved for subsequent offline analysis using CED Signal software (v5.07).

Single-pulse TMS of left M1 was delivered using a MagStim 200 magnetic stimulator (Magstim, Dyfed, UK). A figure-of-eight shaped coil (Magstim D70^2^) was held tangentially to the scalp and perpendicular to the central sulcus, inducing a posterior to anterior current flow in M1. The coil was positioned at the optimal site for eliciting motor evoked potentials (MEPs) in the right BB muscle and the location was marked on the scalp. Active motor threshold (AMT) for the right BB was defined as the minimum stimulus intensity that elicited a MEP in four out of eight trials while performing a sustained weak muscle contraction in the standardized posture at the high support level.

TMS intensity was initially set at 130 % of AMT. The MEP amplitude evoked by this intensity preceding elbow flexion at the high support level (typically around 1 mV) was used as a target for adjusting TMS intensity at other support levels. For adjustment sets, TMS was delivered 150 ms preceding the typical burst onset time every 3–5 repetitions. During the main collection sets, TMS was delivered 50, 100, 150, or 200 ms prior to the typical burst onset time every 4–6 repetitions in a pseudo-randomized order [15]. In total, 64 MEPs were elicited from the right BB at each of the 6 combinations of task and support level. A total of 384 stimulation trials were recorded from each participant.

### Data analysis

EMG traces were inspected for correct task performance and the presence of an appropriate stimulus artifact. Trials that did not meet these criteria were discarded from further analysis. As the primary dependent measure, BB MEP amplitude was measured within a 20 ms window that was determined manually for each participant. Pre-trigger BB activity was measured as the rmsEMG amplitude over a 50 ms window preceding the stimulus.

A task ratio measure was used to quantify the behavior of the task agonist (BB or PT). The ratio was calculated as the rmsEMG amplitude following burst onset, relative to baseline rmsEMG amplitude. An EMG burst onset interval was determined manually for each trace as the time between stimulation and EMG burst onset. Only trials with a burst onset interval between 100–300 ms were included in the analysis. Raw MEP amplitudes were rescaled between 0 and 1 within each participant. Similarly, all rmsEMG values were normalized relative to each participant’s maximum voluntary contraction (MVC) for a given muscle. Stimulus intensity was expressed relative to AMT.

### Statistical analysis

Statistical analyses were carried out using R 3.1.2 [21] with the *nlme*: *Linear and Nonlinear Mixed Effects Models* [22] and *predict means*: *Calculate Predicted Means for Linear Models* packages [23]. Distributional assumptions were examined through inspection of q-q plots.

To examine the effect of weight support on tonic muscle activity, a linear mixed effects analysis was conducted on baseline muscle activity. We modeled support level as a fixed effect and used an error term with random intercepts grouped by subject.

To examine the effect of weight support on corticomotor excitability, a linear mixed effects analysis was conducted on bb mep amplitude with task and support level as categorical factors, and continuous covariates for pre-trigger activity, stimulus intensity, task ratio, and emg burst onset interval. The error term included random intercepts grouped by subject. A random slope was also included for pre-trigger activity. Interpolated means were calculated at the median values of the four covariates.

An alpha level of 0.05 was adopted as the criterion for statistical significance. Post-hoc comparisons were evaluated using Tukey HSD adjusted p-values. Means and standard errors (SE) are reported in the text.

## Results

**Fig. 2:**
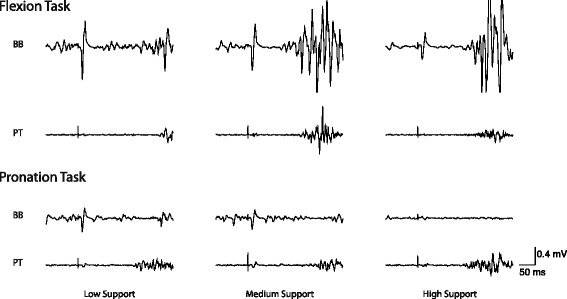
Example EMG traces from a representative participant

None of the 15 participants reported adverse effects from the procedures. Data from 2 participants were not included in the final analysis because of inconsistent task performance as indicated by task ratio values. Of the 64 MEPs collected from each participant per condition, an average of 52 fell within the burst onset interval criteria and were retained for analysis. Example EMG traces are presented in Fig. 2.

### Effect of weight support on tonic muscle activity

**Fig. 3:**
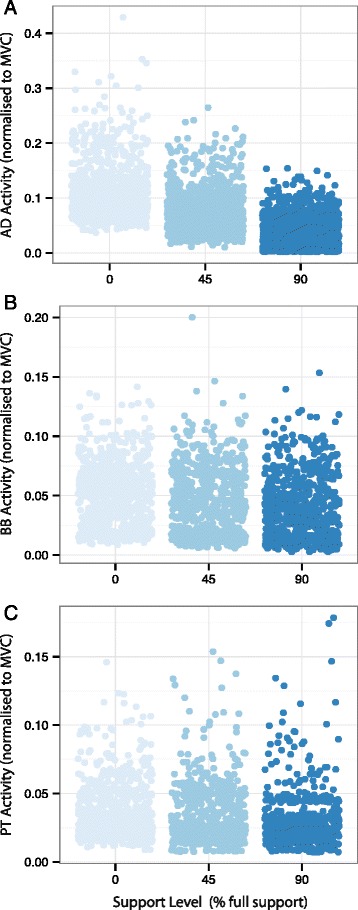
Baseline EMG activity at low (0 %), medium (45 %), and high (90 %) levels of WS is plotted for **a**: *anterior deltoid*, **b**: *biceps brachii*, and **c**: *pronator teres*. Each data point represents rmsEMG from a single trial normalized to maximum voluntary contraction

As expected there was a main effect of support level for AD (F_(2,4017)_ = 2480.37, *p* < 0.0001; Fig 3a), for BB (F_(2,1972)_ = 73.00, *p* < 0.0001; Fig 3b) and for PT (F_(2,2030)_ = 67.70, *p* < 0.0001; Fig 3c). All three muscles exhibited less tonic activity with higher levels of external support. Mean values and standard errors are presented in Table 1.

**Table 1:** Mean (SE) normalized baseline muscle activity expressed as a proportion of maximum voluntary contraction

Muscle	Support level		
	Low (0 %)	Medium (45 %)	High (90 %)
*Anterior deltoid* (AD)	0.1040 (0.0012)	0.0743 (0.0010)	0.0384 (0.0008)
*Biceps brachii* (BB)	0.0508 (0.0010)	0.0448 (0.0011)	0.0397 (0.0011)
*Pronator teres* (PT)	0.0368 (0.0008)	0.0332 (0.0008)	0.0302 (0.0008)

### Effect of weight support on BB MEPs

Omnibus ANOVA results are shown in Table 2 and descriptive statistics in Table 3. There were main effects of task and support level, and a task × support level interaction (all *p* < 0.0001). For covariates, the factors stimulus intensity (*p* = 0.1597) and task ratio (*p* = 0.0741) did not significantly affect bb mep amplitude but were retained in the model. There were significant effects of pre-trigger activity (*p* < 0.0001) and burst onset interval (*p* = 0.0016). MEP amplitude was larger with greater pre-trigger activity and shorter burst onset interval.

**Table 2:** Omnibus ANOVA for linear mixed model of BB MEP amplitude

Factor	DF_Num_	DF_Den_	F	*p*
support level	2	4005	48.69	< .0001
task	1	4005	331.46	< .0001
pre-trigger activity	1	4005	18.53	< .0001
task ratio	1	4005	3.19	0.0741
stimulus intensity	1	4005	1.98	0.1597
burst onset interval	1	4005	9.94	0.0016
support level × task	2	4005	27.33	< .0001
support level × pre-trigger activity	2	4005	6.03	0.0024
task × pre-trigger activity	1	4005	43.11	< .0001
support level × task × pre-trigger activity	2	4005	22.85	< .0001

**Table 3:** Mean (SE) values of observed covariate factors and BB MEP amplitude

Observed variable	Low Support		Medium Support		High Support	
	Flexion	Pronation	Flexion	Pronation	Flexion	Pronation
Pre-trigger activity	0.0415 (0.0010)	0.0303 (0.0009)	0.0361 (0.0010)	0.0254 (0.0008)	0.0311 (0.0010)	0.0169 (0.0007)
Burst onset interval (s)	0.1843 (0.0021)	0.1865 (0.0021)	0.1851 (0.0022)	0.1906 (0.0021)	0.1870 (0.0021)	0.1866 (0.0021)
Stimulus intensity	1.2557 (0.0042)	1.2529 (0.0043)	1.3154 (0.0029)	1.3176 (0.0032)	1.3242 (0.0027)	1.3274 (0.0027)
Task ratio	5.1247 (0.1510)	8.8021 (0.1969)	6.3926 (0.2197)	10.1044 (0.1892)	6.8140 (0.2430)	11.0445 (0.2796)
MEP amplitude	0.3954 (0.0086)	0.2775 (0.0067)	0.3529 (0.0070)	0.2804 (0.0076)	0.3231 (0.0075)	0.1713 (0.0061)

Pre-trigger activity is expressed as a proportion of maximum voluntary contraction. Burst onset interval is expressed in seconds. Stimulus intensity is expressed as a proportion of active motor threshold. Task ratio is an expression of EMG burst amplitude relative to baseline EMG amplitude. Raw MEP amplitudes were rescaled between 0 and 1 within each participant

**Fig. 4:**
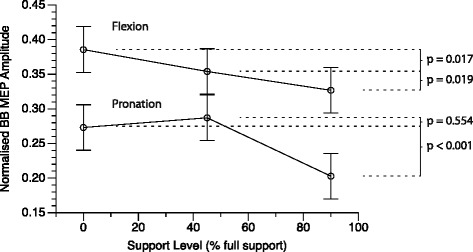
Predicted mean MEP amplitudes for flexion and pronation tasks plotted against WS level. Error bars represent standard error of the mean. Adjusted *p*-values for pairwise comparisons are indicated at right

Because of these covariate effects, it was not possible to perform post-hoc tests on the MEP data directly. Instead, pairwise comparisons were conducted using the linear mixed model to interpolate predicted means at equivalent points along the covariate distributions. Interpolations were made using the following values specified from each covariate distribution: pre-trigger activity of 0.03 × MVC, burst onset interval of 180 ms, stimulus intensity of 1.3 × AMT, and task ratio of 8.0. Predicted means and standard errors for each experimental condition are shown in Fig. 4, and results of pairwise tests in Table 4. For elbow flexion, bb mep amplitude exhibited a negative monotonic relation with support level; MEP amplitude was greater at low support compared to medium support (*p* = 0.017), and likewise greater at medium support than at high support (*p* = 0.019). The omnibus task × support level interaction is apparent in the relation of bb mep amplitude and support level for forearm pronation. MEP amplitude did not differ between low and medium support levels (*p* = 0.554). The hypothesized smaller MEP amplitude with greater WS was observed only at high support (*p* < 0.001).

**Table 4:** Matrix of test statistics for pairwise comparisons of predicted BB MEP amplitude

	low:pron	low:flex	med:pron	med:flex	high:pron	high:flex
low:pron	-	−12.3345	−1.6661	−8.7408	8.0019	−5.9109
low:flex	0.0000	-	10.1635	3.2048	17.9571	5.9573
med:pron	0.5544	0.0000	-	−7.8027	10.5346	−4.8011
med:flex	0.0000	0.0171	0.0000	-	16.8658	3.1792
high:pron	0.0000	0.0000	0.0000	0.0000	-	−14.4229
high:flex	0.0000	0.0000	0.0000	0.0186	0.0000	-

Adjusted *p*-values are below the diagonal, t-statistics are above the diagonal.

## Discussion

This study examined task dependent modulation of *biceps brachii* corticomotor excitability under systematic variation of arm weight support. In line with previous findings, baseline tonic muscle activity decreased across the upper limb as WS was increased. Consistent with our hypothesis, there was improved BB selectivity at the highest level of WS. As an agonist to elbow flexion, BB CME reduced monotonically with each increase in WS. As an antagonist to forearm pronation, BB CME was suppressed only at the highest level of WS. Overall, these results support a model of integrated upper limb control, which interacts with WS through independent excitatory and inhibitory mechanisms. These novel findings may inform applications of WS in clinical settings such as upper limb rehabilitation following stroke.

### Weight support reduces tonic muscle activity across the upper limb

The WS manipulation provides evidence for a common neural drive to muscles of the arm. Greater WS reduced tonic muscle activity across the upper limb. As indicated by baseline EMG values (Table 1, Fig. 3), AD is the principal muscle generating antigravity torque for the posture examined and exhibited the greatest difference in tonic activity between low and high levels of WS. This finding confirms the efficacy of the WS manipulation and is consistent with previous studies employing multiple levels of WS [14, 24]. BB and PT were not positioned to act against gravity, but were recruited primarily during the phasic movement tasks, which did not differ across levels of WS. The tendency for WS to indirectly exert an influence on tonic activity in BB and PT reflects a common drive to muscles across the upper limb.

### Weight support affects task-dependent modulation of corticomotor excitability

*Biceps brachii* MEP amplitude was modulated by the WS manipulation differentially for the flexion and pronation motor tasks. For the elbow flexion task, progressively greater WS resulted in a monotonic decrease in BB MEP amplitude. This observation was made after statistically controlling for variation in pre-trigger EMG activity and EMG burst onset timing and indicates that WS modulates the excitability of neurons upstream of the spinal motor neuron pool. Sensory information from cutaneous mechanoreceptors and muscle spindle afferents differed minimally across levels of WS because posture and bracing were stable. The observed differences in MEP amplitude imply WS modulates motor neuron excitability at a supraspinal level in line with previous evidence for cortically mediated proximal-distal integration [25] and earlier neurophysiological studies of WS [14]. An up-regulation of CME preceding voluntary recruitment of BB for elbow flexion is expected and established in this paradigm as observed previously [15, 16]. The functional significance of weaker BB CME facilitation preceding elbow flexion with WS is not known. The reciprocal ability to suppress CME before an antagonist contraction may be of greater importance to coordination.

Given the role of BB as a forearm supinator, BB CME must be selectively suppressed to pronate the forearm. As hypothesized, BB MEP amplitude was suppressed during the forearm pronation task at the high level of support. This could be indicative of a greater inhibitory network available to suppress CME. A reduction in the activity of proximal anti-gravity muscles may act through putative neural inhibitory networks within M1 to reduce excitatory input to the BB motor neurons. BB MEP amplitude differed between low and medium support during the flexion task, indicating the WS manipulation was able to influence motor cortical excitability under these conditions. The differential pattern of BB CME modulation between flexion and pronation suggests that WS might preferentially engage inhibitory circuits to reduce excitatory activity when antagonist suppression is required. A similar observation was made by Devanne *et al*. [25] who found that shoulder activity could influence short latency intracortical inhibition of M1 forearm representations.

### Neural mechanisms for integrated upper limb control and selective muscle activation

The present findings support a model of integrated neuromuscular activity along the proximal-distal axis. In this study, WS led to changes in tonic muscle activity across the upper limb and a modulation of phasic BB CME. A linked neural architecture may facilitate coordination of behaviors such as forward reaching and could incorporate cortical and subcortical mechanisms. A cortical basis for proximal-distal linkages is supported by evidence of multiple non-contiguous muscle representations in the animal M1 that exhibit extensive overlap with those of other muscles [26–28]. Furthermore, representations of proximal muscles have been reported to systematically surround those of more distal muscles [29]. A similar overlapping architecture has been observed in the human motor cortex [30, 31]. In the context of present findings, the direct effect of WS on voluntary drive to the AD could propagate to linked representations and result in positively correlated changes in tonic motor neuron activity.

The linking of muscle activity across the upper limb may also be mediated *via* subcortical and spinal mechanisms. Extensive divergence of descending corticomotor pathways could contribute a common obligatory drive to multiple motor neuron pools [32]. In parallel, ipsilateral descending pathways contribute to proximal limb control and exhibit less specific patterns of innervation. Notably, suppression of the ipsilateral motor cortex by cathodal transcranial direct current stimulation mediates an improvement in BB selectivity [33, 34]. Other motor pathways such as the C3-C4 propriospinal system link multiple spinal segments and can modulate cortical output to the forearm [35, 36]. Additionally, spinal interneuron circuits can produce stable patterns of coordinated activity across multiple muscles [37, 38]. In summary, it is likely that a combination of cortical and subcortical mechanisms contributes to a scaling of tonic activity across the upper limb with changes in WS.

A task dependent modulation of phasic CME suggests that WS interacts with excitatory linking mechanisms as well as local inhibitory circuits. The monotonic pattern of CME modulation preceding elbow flexion indicates a linear scaling of excitatory inputs, whereas the pattern preceding pronation may be indicative of a threshold or saturation effect in the inhibitory circuits. Selective recruitment of upper limb muscles like BB requires centrally mediated phasic activation commands that may be superimposed upon tonic co-activation [39, 40]. Although agonist–antagonist pairs are reciprocally inhibited at the spinal level, sophisticated activation patterns are possible because cortical representations are linked through excitatory axon collaterals [41]. The action of local inhibitory circuits normally suppresses cortical motor neurons but can be lifted either selectively or in concert, to achieve the desired pattern of motor output [42]. The differential response of phasic CME is indicative that WS interacts with local inhibitory circuits independently of excitatory linking mechanisms.

### Potential limitations

A limitation of the present study is an absence of behavioral data beyond the EMG measures. Whether there are functional correlates of the observed CME modulation is not clear. Notably there was no clear trend or statistically significant effect of the EMG-based task ratio measure. Furthermore this study sampled young healthy adults, a population that other studies have reported is able to maintain invariant reaching kinematics with changes in WS [12, 24]. Future studies are warranted to examine the effects of WS on CME and arm function in neurologically impaired individuals (e.g. after stroke resulting in upper limb impairment). It is clear that reduced suppression of BB CME preceding an antagonist contraction is related to upper limb impairment in stroke patients [16, 18].

### Implications for the use of weight support for neurorehabilitation

Weight support of the arm may have relevance to upper limb rehabilitation after brain injuries such as stroke [1–3]. In addition to facilitating training dosage, WS may facilitate the production of movements that are not achievable without assistance. By reducing the amount of muscle activity required to move the paretic upper limb, WS can reduce the effect of abnormal joint coupling and increase the reachable workspace area [5, 11, 43–46]. This could be advantageous for practicing reach and retrieval tasks that are associated with daily living activities such as feeding, grooming, dressing, or preparing meals. It remains to be determined if the extent to which WS may improve the selective activation of muscles like BB depends on structures affected, such as extent of damage to motor cortex, or the corticospinal tract. We previously reported that in healthy adults, WS influences CME across the upper limb [14]. This study demonstrates additionally that WS modulates CME during a phasic movement task, improving BB selectivity. In the context of integrated limb control, the level of WS may interact with excitatory and inhibitory mechanisms independently. Independent modulation could in turn create unique neuromechanical control profiles and opportunities for targeted therapeutic exercises. By facilitating otherwise unachievable patterns of neuromuscular activity in addition to training dosage, WS may be a valuable tool for driving neuroplasticity. Moreover, the use of progressive loading with partial rather than full WS may in itself be an important factor driving recovery of upper limb function [47]. Further characterizing muscle coordination and control at different levels of WS [24] will contribute to optimizing the use of WS as an adjuvant to upper limb rehabilitation therapy.

## Conclusions

A manipulation of WS led to changes in tonic muscle activity across the upper limb and a task-dependent modulation of phasic corticomotor excitability to *biceps brachii*. For elbow flexion, corticomotor excitability to *biceps brachii* was reduced with incremental increases in WS. For forearm pronation, corticomotor excitability to *biceps brachii* was reduced only with high WS. This different pattern of modulation indicates WS interacts with inhibitory circuits independently, potentially increasing the inhibitory network available to suppress unwanted muscle recruitment. Overall, these results demonstrate that the amount of WS has direct and indirect influences on neuromuscular activity across the upper limb. Tunable supportive force may be an important consideration in the design and application of WS devices. With further characterization of parametric WS, its role in neurorehabilitation may be refined and individualized.

## Abbreviations

AD: Anterior deltoid
AMT: Active motor threshold
BB: Biceps brachii
CME: Corticomotor excitability
M1: Primary motor cortex
MEP: Motor-evoked potential
MVC: Maximum voluntary contraction
PT: Pronator teres
rmsEMG: Root mean squared electromyogram
SE: Standard error
TMS: Transcranial magnetic stimulation
WS: Weight support of the arm

## References

[1] Prange GB, Jannink MJA, Groothuis-Oudshoorn CGM, Hermens HJ, Ijzerman MJ (2006). Systematic review of the effect of robot-aided therapy on recovery of the hemiparetic arm after stroke. J Rehabil Res Dev.

[2] Brewer BR, McDowell SK, Worthen-Chaudhari LC (2007). Poststroke upper extremity rehabilitation: a review of robotic systems and clinical results. Top Stroke Rehabil.

[3] Mehrholz J, Hädrich A, Platz T, Kugler J, Pohl M (2012). Electromechanical and robot-assisted arm training for improving generic activities of daily living, arm function, and arm muscle strength after stroke. Cochrane Database Syst Rev.

[4] Loureiro RCV, Harwin WS, Nagai K, Johnson M (2011). Advances in upper limb stroke rehabilitation: a technology push. Med Biol Eng Comput.

[5] Sukal TM, Ellis MD, Dewald JPA (2007). Shoulder abduction-induced reductions in reaching work area following hemiparetic stroke: neuroscientific implications. Exp Brain Res.

[6] Kwakkel G, Meskers CGM (2014). Effects of robotic therapy of the arm after stroke. Lancet Neurol.

[7] Nudo RJ, Milliken GW, Jenkins WM, Merzenich MM (1996). Use-dependent alterations of movement representations in primary motor cortex of adult squirrel monkeys. J Neurosci.

[8] Woldag H, Hummelsheim H (2002). Evidence-based physiotherapeutic concepts for improving arm and hand function in stroke patients: a review. J Neurol.

[9] Kleim JA, Jones TA (2008). Principles of experience-dependent neural plasticity: implications for rehabilitation after brain damage. J Speech Lang Hear Res.

[10] Dewald JPA, Beer RF (2001). Abnormal joint torque patterns in the paretic upper limb of subjects with hemiparesis. Muscle Nerve.

[11] Beer RF, Dewald JPA, Dawson ML, Rymer WZ (2004). Target-dependent differences between free and constrained arm movements in chronic hemiparesis. Exp Brain Res.

[12] Prange GB, Kallenberg LAC, Jannink MJA, Stienen AHA, van der Kooij H, IJzerman MJ (2009). Influence of gravity compensation on muscle activity during reach and retrieval in healthy elderly. J Electromyogr Kinesiol.

[13] Prange GB, Jannink MJA, Stienen AHA, van der Kooij H, IJzerman MJ, Hermens HJ (2009). Influence of Gravity Compensation on Muscle Activation Patterns During Different Temporal Phases of Arm Movements of Stroke Patients. NNR.

[14] Runnalls KD, Anson G, Wolf SL, Byblow WD (2014). Partial weight support differentially affects corticomotor excitability across muscles of the upper limb. Physiol Rep.

[15] Gerachshenko T, Stinear JW (2007). Suppression of motor evoked potentials in biceps brachii preceding pronator contraction. Exp Brain Res.

[16] Gerachshenko T, Rymer WZ, Stinear JW (2008). Abnormal corticomotor excitability assessed in biceps brachii preceding pronator contraction post-stroke. Clin Neurophysiol.

[17] Bradnam LV, Stinear CM, Byblow WD (2010). Theta burst stimulation of human primary motor cortex degrades selective muscle activation in the ipsilateral arm. J Neurophysiol.

[18] Bradnam LV, Stinear CM, Barber PA, Byblow WD (2012). Contralesional hemisphere control of the proximal paretic upper limb following stroke. Cereb Cortex.

[19] Bradnam LV, Stinear CM, Byblow WD (2013). Ipsilateral motor pathways after stroke: implications for non-invasive brain stimulation. Front Hum Neurosci.

[20] Dewald JPA, Pope PS, Given JD, Buchanan TS, Rymer WZ (1995). Abnormal muscle coactivation patterns during isometric torque generation at the elbow and shoulder in hemiparetic subjects. Brain.

[21] R Core Team (2014). R: A Language and Environment for Statistical Computing.

[22] Pinheiro J, Bates D, R-core Team (2015). nlme: Linear and Nonlinear Mixed Effects Models.

[23] Luo D, Ganesh S, Koolaard J (2014). predictmeans: Calculate Predicted Means for Linear Models.

[24] Coscia M, Cheung VCK, Tropea P, Koenig A, Monaco V, Bennis C (2014). The effect of arm weight support on upper limb muscle synergies during reaching movements. J NeuroEngineering Rehabil.

[25] Devanne H, Cohen LG, Kouchtir-Devanne N, Capaday C (2002). Integrated motor cortical control of task-related muscles during pointing in humans. J Neurophysiol.

[26] Donoghue JP, Leibovic S, Sanes JN (1992). Organization of the forelimb area in squirrel monkey motor cortex: representation of digit, wrist, and elbow muscles. Exp Brain Res.

[27] Schneider C, Zytnicki D, Capaday C (2001). Quantitative evidence for multiple widespread representations of individual muscles in the cat motor cortex. Neurosci Lett.

[28] Rathelot JA, Strick PL (2006). Muscle representation in the macaque motor cortex: an anatomical perspective. Proc Natl Acad Sci USA.

[29] Park MC, Belhaj-Saif A, Gordon M, Cheney PD (2001). Consistent features in the forelimb representation of primary motor cortex in rhesus macaques. J Neurosci.

[30] Sanes JN, Donoghue JP, Thangaraj V, Edelman RR, Warach S (1995). Shared neural substrates controlling hand movements in human motor cortex. Science.

[31] Devanne H, Cassim F, Ethier C, Brizzi L, Thevenon A, Capaday C (2006). The comparable size and overlapping nature of upper limb distal and proximal muscle representations in the human motor cortex. Eur J Neurosci.

[32] McKiernan BJ, Marcario JK, Karrer JH, Cheney PD (1998). Corticomotoneuronal postspike effects in shoulder, elbow, wrist, digit, and intrinsic hand muscles during a reach and prehension task. J Neurophysiol.

[33] McCambridge AB, Bradnam LV, Stinear CM, Byblow WD (2011). Cathodal transcranial direct current stimulation of the primary motor cortex improves selective muscle activation in the ipsilateral arm. J Neurophysiol.

[34] Uehara K, Coxon JP, Byblow WD (2015). Transcranial direct current stimulation improves ipsilateral selective muscle activation in a frequency dependent manner. PLoS One.

[35] Pauvert V, Pierrot-Deseilligny E, Rothwell JC (1998). Role of spinal premotoneurones in mediating corticospinal input to forearm motoneurones in man. J Physiol Lond.

[36] Pierrot-Deseilligny E (2002). Propriospinal transmission of part of the corticospinal excitation in humans. Muscle Nerve.

[37] Bizzi E, Mussa-Ivaldi FA, Giszter S (1991). Computations underlying the execution of movement: a biological perspective. Science.

[38] Bizzi E, Cheung VCK (2013). The neural origin of muscle synergies. Front Comput Neurosci.

[39] De Luca CJ, Mambrito B (1987). Voluntary control of motor units in human antagonist muscles: coactivation and reciprocal activation. J Neurophysiol.

[40] Flanders M, Herrmann U (1992). Two components of muscle activation: scaling with the speed of arm movement. J Neurophysiol.

[41] Capaday C, Devanne H, Bertrand L, Lavoie BA (1998). Intracortical connections between motor cortical zones controlling antagonistic muscles in the cat: a combined anatomical and physiological study. Exp Brain Res.

[42] Ethier C, Brizzi L, Giguère D, Capaday C (2007). Corticospinal control of antagonistic muscles in the cat. Eur J Neurosci.

[43] Beer RF, Ellis MD, Holubar BG, Dewald JPA (2007). Impact of gravity loading on post-stroke reaching and its relationship to weakness. Muscle Nerve.

[44] Ellis MD, Sukal-Moulton TM, Dewald J (2009). Impairment-Based 3-D Robotic Intervention Improves Upper Extremity Work Area in Chronic Stroke: Targeting Abnormal Joint Torque Coupling With Progressive Shoulder Abduction Loading. IEEE Trans Robot.

[45] Housman SJ, Scott KM, Reinkensmeyer DJ (2009). A randomized controlled trial of gravity-supported, computer-enhanced arm exercise for individuals with severe hemiparesis. NNR.

[46] Krabben T, Prange GB, Molier BI, Stienen AHA, Stienen AH, Jannink MJ (2011). Influence of gravity compensation training on synergistic movement patterns of the upper extremity after stroke, a pilot study. J NeuroEngineering Rehabil.

[47] Ellis MD, Sukal-Moulton T, Dewald JPA (2009). Progressive shoulder abduction loading is a crucial element of arm rehabilitation in chronic stroke. NNR.

